# Low Temperature and Cold Stress Significantly Increase Saxitoxins (STXs) and Expression of STX Biosynthesis Genes *sxtA4* and *sxtG* in the Dinoflagellate *Alexandrium catenella*

**DOI:** 10.3390/md19060291

**Published:** 2021-05-21

**Authors:** Hansol Kim, Hyunjun Park, Hui Wang, Hah Young Yoo, Jaeyeon Park, Jang-Seu Ki

**Affiliations:** 1 Department of Biotechnology, Sangmyung University, Seoul 03016, Korea; 201934001@sangmyung.kr (H.K.); 202032006@sangmyung.kr (H.P.); 201534008@sangmyung.kr (H.W.); y2h2000@smu.ac.kr (H.Y.Y.); 2 Environment and Resource Convergence Center, Advanced Institute of Convergence Technologies, Suwon 16229, Korea

**Keywords:** *Alexandrium catenella*, saxitoxins (STXs), saxitoxin biosynthesis genes, temperature, transcriptional response

## Abstract

Toxic dinoflagellate *Alexandrium* spp. produce saxitoxins (STXs), whose biosynthesis pathway is affected by temperature. However, the link between the regulation of the relevant genes and STXs’ accumulation and temperature is insufficiently understood. In the present study, we evaluated the effects of temperature on cellular STXs and the expression of two core STX biosynthesis genes (*sxtA4* and *sxtG*) in the toxic dinoflagellate *Alexandrium* *catenella* Alex03 isolated from Korean waters. We analyzed the growth rate, toxin profiles, and gene responses in cells exposed to different temperatures, including long-term adaptation (12, 16, and 20 °C) and cold and heat stresses. Temperature significantly affected the growth of *A. catenella*, with optimal growth (0.49 division/day) at 16 °C and the largest cell size (30.5 µm) at 12 °C. High concentration of STXs eq were detected in cells cultured at 16 °C (86.3 fmol/cell) and exposed to cold stress at 20→12 °C (96.6 fmol/cell) compared to those at 20 °C and exposed to heat stress. Quantitative real-time PCR (qRT-PCR) revealed significant gene expression changes of *sxtA4* in cells cultured at 16 °C (1.8-fold) and cold shock at 20→16 °C (9.9-fold). In addition, *sxtG* was significantly induced in cells exposed to cold shocks (20→16 °C; 19.5-fold) and heat stress (12→20 °C; 25.6-fold). Principal component analysis (PCA) revealed that low temperature (12 and 16 °C) and cold stress were positively related with STXs’ production and gene expression levels. These results suggest that temperature may affect the toxicity and regulation of STX biosynthesis genes in dinoflagellates.

## 1. Introduction

Dinoflagellates are unicellular micro-eukaryotes that mostly inhabit marine water, with some observed in freshwater. They have evolved to adapt to different environments with a variety of morphological diversity and trophic modes [1]. Approximately half of the dinoflagellates are photosynthetic; thus, as a primary producer, they play an important role in aquatic ecosystems [2]. Some dinoflagellates, however, are responsible for harmful algal blooms (HABs), causing considerable damages to marine environments and aquaculture, and even human health [3,4,5].

In addition, certain dinoflagellates (e.g., *Alexandrium catenella*, *Gymnodinium catenatum*, *Karenia brevis*, and *Prorocentrum minimum*) can produce biotoxins, such as neurotoxins and hepatotoxins, and these compounds can be accumulated in shellfish via filter feeding [6,7,8]. Thus, toxic dinoflagellates can cause shellfish poisoning when humans consume contaminated shellfish. Approximately 2000 cases of shellfish poisoning are reported annually around the world, resulting in serious illness or even death [9]. There are four typical types of shellfish poisonings, viz., amnesic shellfish poisoning (ASP), diarrhetic shellfish poisoning (DSP), neurotoxic shellfish poisoning (NSP), and paralytic shellfish poisoning (PSP). Among these, PSP is the most serious syndrome reported worldwide, presenting both gastrointestinal and neurological symptoms [10].

Saxitoxin (STX) and its analogues (STXs) are neurotoxins that are naturally produced by certain species of marine and freshwater phytoplankton. STX analogues such as gonyautoxins (GTX1-6), N-sulfocarbamoylgonyautoxins (C1-2), decarbamoylgonyautoxins (dcGTX1-4), STX, neosaxitoxin (neoSTX) and decarbamoylsaxitoxin (dcSTX) are referred to as paralytic shellfish toxins (PSTs). These substances reversibly bind the voltage-gated Na+ channels of neurons, causing paralysis [11,12] and are 2000-times more toxic than sodium cyanide [13]. STXs’ accumulation in bivalve filter feeders is well-known and was first described in clam and mussel tissues in 1957 [14]. In marine environments, STXs are primarily produced by toxic dinoflagellates including several *Alexandrium* species, *G. catenatum*, and *Pyrodinium bahamense* [5,15,16].

Cell growth and cellular STXs’ content in the toxic *Alexandrium* species vary according to environmental factors, such as temperature, salinity, light intensity, CO_2_, and nutrients [17,18,19]. As eutrophication is responsible for HABs, effects of nutrients on STX biosynthesis have been extensively studied in toxic cyanobacteria and marine dinoflagellates until now [17,20,21]. In addition, STXs accumulated in shellfish are detected in spring and autumn off the coasts of temperate regions, including western Mediterranean, Chile, New Zealand, and Korea [22,23,24,25]. Vandersea et al. [26] pointed out that the abundance of *A. catenella* and STXs’ production may be more related to water temperature than to nutrients or salinity. Although controversial, environmental surveys and laboratory experiments suggest that temperature may affect STXs’ production in toxic *Alexandrium* [27,28,29]. It is also confusing whether it is due to cellular biomass changes or triggering of STX biosynthesis [17,19,30]. Hence, to understand the influence of abiotic factors at the molecular level, research on STX synthesis genes is necessary.

STX biosynthesis gene clusters were first identified in the freshwater cyanobacterium *Cylindrospermopsis raciborskii* [31], followed by *Anabaena circinalis*, *Aphanizomenon gracile* [32] and *Dolichospermum circinale* [33]. STX biosynthetic pathways have been proposed in cyanobacteria [32]. These studies have identified at least 26 enzymes (e.g., sxtA, sxtG, sxtB, sxtC, sxtD, and sxtH/T) involved in the biosynthesis, tailoring, transport, and/or regulation of STXs. These findings suggested that toxic dinoflagellates seem to harbor a similar system for STX biosynthesis. Thus, considering STX biosynthesis genes in toxic cyanobacteria, many researchers have explored their homologs and orthologs in marine dinoflagellates using gene cloning, transcriptomics, and synthetic pathway analysis [3,34,35]. In contrast to cyanobacteria, molecular studies on STX-producing dinoflagellates are limited and are further complicated owing to their extraordinary genetic characteristics, including a huge genome, permanently condensed chromosomes, high GC content, and post-transcriptional regulation [36]. Recent large-scale transcriptomic analyses using next-generation sequencing (NGS) have enabled the characterization and identification of homologous genes in toxic *Alexandrium* [37,38,39,40,41,42,43].

STX biosynthesis begins with a polyketide-like synthase, catalyzing arginine and malonyl-CoA; this is followed by another reaction catalyzed by amidinotransferase that transfers the amidino group from arginine [44]. sxtA enzyme comprises four catalytic domains, and the enzyme serves as S-adenosyl-methionine (SAM)-dependent methyltransferase (sxtA1), GCN5-related N-acetyltransferase (sxtA2), acyl carrier protein (sxtA3), and a class II aminotransferase (sxtA4). Particularly, sxtA4 is important for STX biosynthesis because it is not present in non-toxic dinoflagellates [31,40,41,45,46]. In addition, our recent transcriptomic analysis showed that the toxic *Alexandrium* spp. contain most of the core STX biosynthesis genes (*sxtA*, *sxtB*, *sxtD*, *sxtG*, *sxtH*/*T*, *sxtI*, *sxtS*, and *sxtU*), whereas their structural modifications and/or absence were observed in non-toxic dinoflagellates [41,42]. Of these genes, *sxtA* and *sxtG* are involved in the initial steps of STX biosynthesis and have been investigated at transcriptomic and genomic levels [45,47]. To date, these genes are considered to play the most important role in STX biosynthesis [48]; however, the relationship between STX toxicity and response of the core genes to environmental factors is insufficiently understood.

In the present study, we evaluated the effects of temperature on cellular STXs’ accumulation and expression of the two core STX biosynthesis genes, *sxtA4* and *sxtG,* in the toxic dinoflagellate *Alexandrium catenella* (Alex03). Then, we compared the relationship between temperature and STX production through transcriptional regulation. The test species occurs predominantly in Korean coasts and is suspected to cause PSP in marine aquaculture thorough molecular phylogeny and laboratory culture researches [49,50].

## 2. Results

### 2.1. Effects of Temperature on Cell Growth and Size

**Figure 1:**
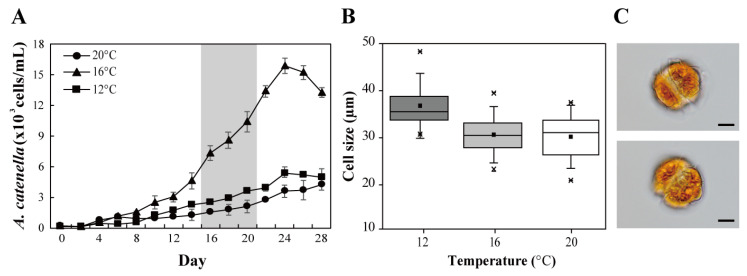
Growth curves (**A**) and box plots of cell size (**B**) of *Alexandrium catenella* Alex03 dependent on adapted temperature (12, 16, and 20 °C). The “*” were determined by the 1st and 99th percentiles. Morphology of *A. catenella* observed in bright-field microscopy (scale bar: 20 µm) (**C**). Cell growth was observed over a period of 28 days, and the gray background in growth curve represents the presence of an exponential phase. The size of the cells harvested on day 18, i.e., the exponential phase was analyzed.

*Alexandrium catenella* Alex03 was cultured at 20 °C, and was successively adapted to different temperatures (16 °C and 12 °C). Cells cultured at lower temperatures were healthy and not much different compared to those cultured at 20 °C. Cell growth patterns in the three test temperatures showed sigmoidal cell growth at 16 °C but relatively slow growth at 12 °C and 20 °C (Figure 1A). Comparisons at the exponential phase (day 18) revealed that cell number at 16 °C was much higher than that at 12 °C (2.9-times at 16 °C) and 20 °C (4.7-times at 16 °C). Hence, optimal cell growth was recorded at 16 °C, with the maximum cell density of 5.9 × 10^3^ cells/mL, of which growth rate was calculated to be 0.49 division/day.

The average cell size of three cultures was the largest at 12 °C (36.7 ± 0.5 µm), followed by those at 16 °C (30.7 ± 0.3 µm) and 20 °C (30.5 ± 0.5 µm) (Figure 1B). The widest distribution of cell size was recorded at 20 °C, ranging from 23.1 to 37.4 µm.

### 2.2. Phylogenetic Relationships of sxtA4 and sxtG and Characterization

Partial open reading frame (ORF) sequences of *sxtA4* and *sxtG* from *A. catenella* were determined to be 714 bp and 1112 bp, respectively. BLASTx search of the *sxtA4* sequence of Alex03 (GenBank accession no. MW884259) showed 100% identity with that of the dinoflagellate *A. fundyense* (ADY62525), followed by 99.5% with *A. tamarense* (BCG06333) and 96.3% with *A. australiense* (AIY25738). The *sxtG* sequence (MW884258) of Alex03 matched 100% with the same gene of *A. tamarense* (AGC84356), 99.7% with *A. fundyense* (AGC84339.1), and 96.3% with *A. insuetum* (AGC84351). In silico analyses of functional domains predicted that a class II aminotransferase was present at 23 to 226 amino acid residue of *sxtA4* protein, whereas no specific domain was found in *sxtG* of Alex03.

**Figure 2:**
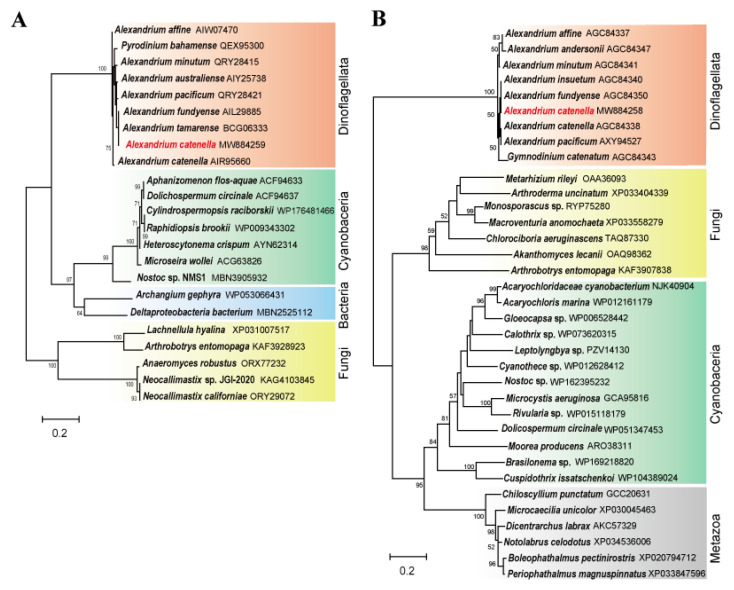
Phylogenic trees of *sxtA4* (**A**) and *sxtG* (**B**). The tree was separately constructed with deduced amino acid sequences of sxtA4 and sxtG using maximum likelihood (ML) method. The sequences of *Alexandrium catenella* Alex03 are marked in red. GenBank accession numbers are given after species name.

In addition, we investigated phylogenetic relationships of the genes *sxtA4* and *sxtG* using broad taxon samplings from NCBI. ML trees of the deduced amino acid sequences showed that *A. catenella* Alex03 obviously formed one well-supported cluster, with the already known *Alexandrium* spp. (Figure 2). In particular, the phylogenetic tree of *sxtA4* showed that the gene was clustered with *A. fundyense* and *A. tamarense*, and it formed a sister clade, which was separated into cyanobacteria and bacteria. In addition, sxtG protein also clustered into a single clade, which included other *Alexandrium* species (*A. catenella*, *A. fundyense*, *A. insuetum*) and *G. catenatum*. The sxtG clade was divided into two sub-clusters of fungi and the sister clades of cyanobacteria and metazoa.

### 2.3. Effect of Water Temperature on STXs

**Figure 3:**
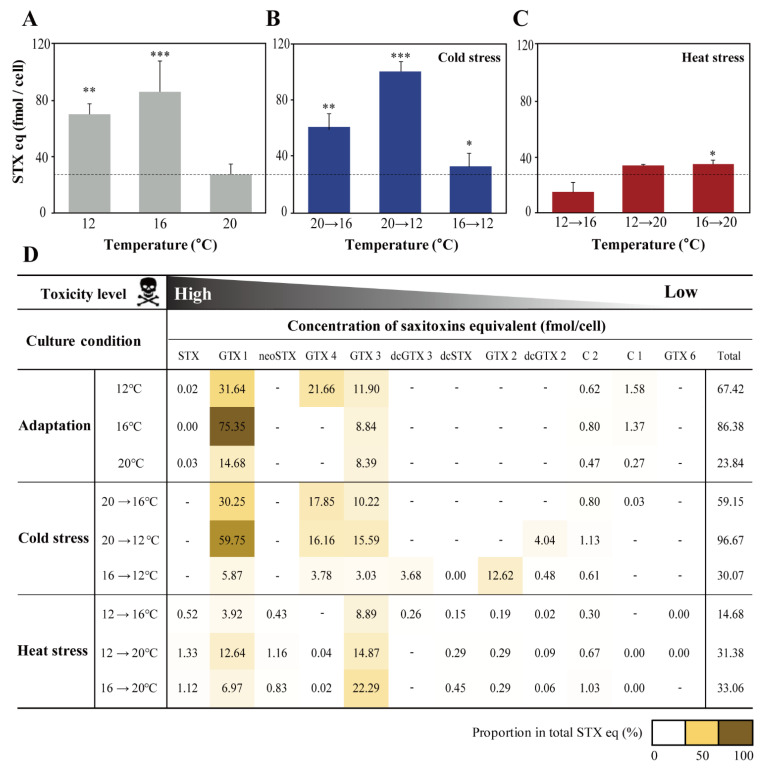
Comparison of saxitoxins equivalent (STXs eq) of *Alexandrium catenella* Alex03 in different temperatures; adaptation temperature (**A**), cold stress (**B**), and heat stress (**C**). The mean STXs eq (fmol/cell) for each STX analogues and total STXs eq were calculated and displayed to two decimal places. Undetected analogues were marked ‘-’. The proportion of each STX analogues (%) to the total STXs eq is expressed by a heatmap (**D**). Significant differences between the control and treated samples were determined by one-way ANOVA and highlighted as * *p* < 0.05, ** *p* < 0.01, and *** *p* < 0.001.

Total STXs eq for each sample was determined by summing 12 STX analogues calculated with toxicity equivalency factors (TEF). The results showed that temperature significantly changed the total STXs eq and their profiles in *A. catenella* (Figure 3). STXs eq was the highest at 16 °C (86.38 fmol/cell), which was 3.6- and 1.2-times higher than that at 20 °C (*p <* 0.001) and 12 °C (*p* < 0.01), respectively. In addition, total STXs eq increased significantly when exposed to 20→12 °C (96.67 fmol/cell; *p* < 0.001), followed by 20→16 °C (59.15 fmol/cell; *p* < 0.01) and 16→12 °C (30.07 fmol/cell; *p* < 0.05). Interestingly, there was no significant changes in STXs eq of heat stress samples, including 16→20 °C (33.06 fmol/cell), 12→20 °C (31.38 fmol/cell), and 12→16 °C (14.68 fmol/cell) compared to that of 20 °C.

The STXs’ profiles of *A. catenella* exposed to different temperatures and cold/heat stress were compared (Figure 3D). Of the 12 STXs, GTX1, GTX3, and GTX4 were most dominantly detected in Alex03. Particularly, GTX1 accounts for the highest proportion of total STXs eq, which is up to 87.2% at 16 °C and 61.8% at 20→12 °C. GTX4 then constitutes 32.1% at 12 °C and 30.1% at 20→16 °C. In addition, GTX3 accounted up to 67.4% at 16→20 °C. STX, dcSTX, neoSTX, and GTX2 were mainly detected in heat shock samples and the others were negligibly found (less than 5%), except for dcGTX3 (12.2%) and GTX2 (41.9%) in 16→12 °C.

### 2.4. Effects of Different Temperatures on sxtA4 and sxtG Transcription

**Figure 4:**
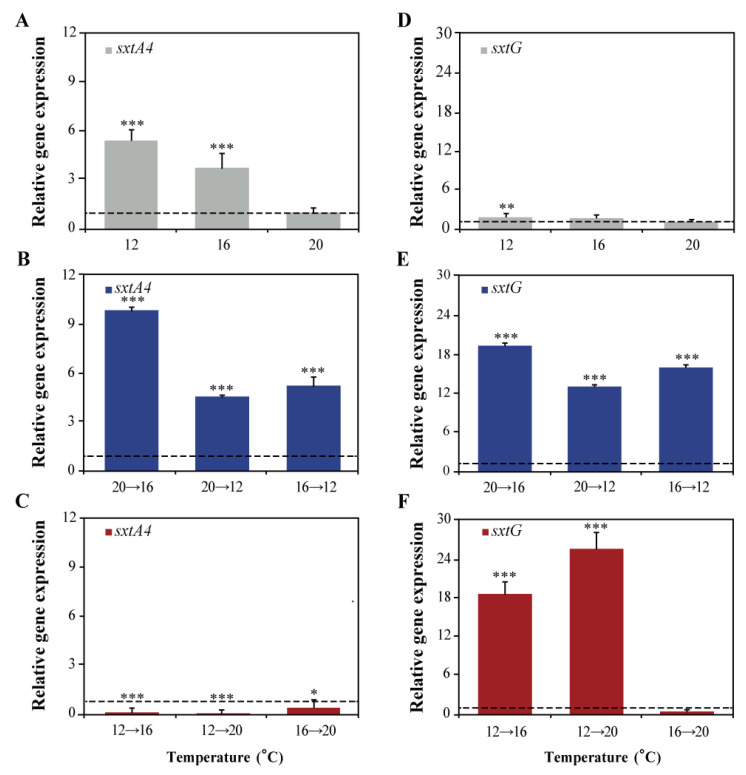
Changes in expression levels of *sxtA4* (**A**–**C**) and *sxtG* (**D**–**F**) dependent on culture conditions with different temperature. *α-tublin* (*TUA*) was used to normalize between different samples. Significant differences between the control and treated samples were determined by one-way ANOVA and highlighted as * *p* < 0.05, ** *p* < 0.01, and *** *p* < 0.001.

The relative expression levels of the genes, *sxtA4* and *sxtG*, were evaluated in *A. catenella* Alex03 exposed to different temperatures for 72 h (Figure 4). The relative expressional levels of each gene were normalized using *TUA*. Expressional levels of *sxtA4* were significantly upregulated at 12 °C (5.4-fold), 16 °C (3.7-fold), and cold stresses (9.9-fold changes at 20→16 °C, 5.2-fold at 16→12 °C, and 4.5-fold at 20→12 °C) compared to control (20 °C). Heat stress, however, significantly reduced *sxtA4* expression levels (0.1-fold change at 12→16 °C and 0.08-fold at 12→20 °C; *p* < 0.001). In addition, relative expression levels of *sxtG* significantly increased when subjected to temperature changes (19.5-, 16.2-, 13.3-, 18.6-, and 25.6-fold at 20→16 °C, 16→12 °C, 20→12 °C, 12→16 °C, and 12→20 °C, respectively) compared to control (20 °C).

### 2.5. Correlation of Temperature, STXs eq and Sxt Genes Expression

**Figure 5:**
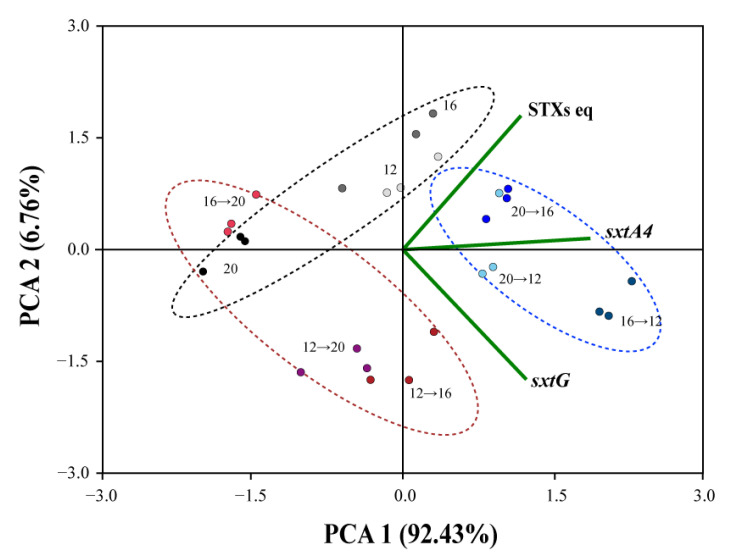
Principal component analysis (PCA) of temperature parameters (adaptation temperature, cold stress, and heat stress) for saxitoxins equivalent (STXs eq) and relative expression levels of STX biosynthesis-related genes (*sxtA4* and *sxtG*).

PCA analysis showed a distinct partitioning of heat and cold stress samples and correlation with total STXs eq, *sxtA4* and *sxtG* expression levels (Figure 5). The ordination plot showed 99.19% of total variation in the data, and a linear positive relation was shown between STXs eq toxicity, *sxtA4*, and *sxtG*. PCA1 explained 92.43% of the variance, whereas PCA2 explained 6.76% of the variance. The samples cultured at 20 °C, 16→20 °C, and 12→20 °C (heat stress) were well-represented on the negative part of PCA1, with no strong relation between STXs eq, *sxtA4*, and *sxtG*. Conversely, cold stress samples (20→16 °C, 20→12 °C and 16→12 °C) and those cultured at 12 °C and 16 °C were clustered in the positive portion of PCA1. Strong affinity was observed between cold stress and STXs eq and expressional levels of the genes.

## 3. Discussion

*A. catenella* is known for producing STXs, but its toxicity varies depending on geographical origin and environmental conditions, even for cultured strains [4,51,52]. *A. catenella* Alex03 was isolated in 2017 from Jeju Island coasts, Korea. Although it had been cultured for a long time and adapted well to laboratory conditions, we found that the morphology and STXs’ toxicity may not or were lightly altered. In addition, comparisons of the 28S rRNA sequences showed that *A. catenella* Alex03 (GenBank accession no. MW882944) shared 100% similarity with *A. catenella* CCAP-1119/32 (MK566200; isolated from Scotland), H5 (MK566199; Argentina), and SCCAP K-1490 (MK566199; Canada). These results suggested that Alex03 was genetically similar or identical to *A. catenella* distributed in coastal waters around the world. The present research firstly evaluated STXs’ production of *A. catenella* Alex03 isolated from Korean coast and analyzed the regulation of *sxt* genes under different temperature conditions.

Molecular phylogeny using 28S rRNA showed that *A. catenella* Alex03 belonged to Group I (=*A. fundyense*) of *A. tamarense* complex [53,54], of which members are known as STX-producing *Alexandrium* [16,55]. Alex03 produced different levels of STXs in all culture conditions. In addition, its STXs eq toxicity (14–96 fmol/cell) was comparable to those reported previously in *A. catenella* (Table 1). The test strain (Alex03) showed optimal growth at 16 °C, in agreement with previous reports [56,57,58]. For examples, *A. catenella* CCAP-1119/27, ATTL01 and ATTL02, and BAH91 contained 2732.5 fg/cell, 5–44 fg/cell, and 9.9 fmol/cell when maintained at 15 °C. In addition, *A. catenella* ACC02 maintained at low temperature (10 °C) were found to be the most toxic (27.7 fmol/cells) [56]. These results reveal that the high toxicity of *A. catenella* may be related to optimal growth temperature at around 16 °C. Moreover, the difference in STXs’ toxicity may be caused by the components of STXs, in which STX and GTX1 were much more toxic than C1 and C2 [59]. *A. catenella* ACT03 showed different dominant analogues depending on culturing temperatures, for example, C1 at 12 °C and GTX4 at 18–30 °C [58]. These results were well-matched with that of Alex03, in which GTX1 was dominantly analyzed at 16 °C.

The STXs’ toxicity of *A. catenella* was further compared to other toxic *Alexandrium* species according to cultured temperatures. It was revealed that *Alexandrium* sp., which belongs to the *A. tamarense* complex, can produce STXs at optimal growth temperature and lower than 20 °C [63,64]. For example, *A. fundyense* BOF and MI were most toxic (100–544 fmol/cell) at 5 °C, and GTX 1–4 and STX with high TEF were primarily detected [61]. In addition, *A. tamarense* contained variants of GTX 1–4 at 15 °C and 17 °C, while the highest toxicity at 12 °C was analyzed [64]. Moreover, when *Alexandrium minutum* was incubated over a wide temperature range (15–25 °C), GTX3–5 were predominantly detected at low temperature [20]. In contrast, *A. affine* and *A. andersonii* were cultured at high temperature (15–34 °C) without producing STXs. These results and our finding showed that the toxic *Alexandrium* spp. vary their STX levels and profiles depending on the strains and incubating temperatures [4,51,52]. In addition, total STXs eq and components may be affected by culture temperature, with the highest toxicity under optimal growth condition [19].

Cell growth and STX toxicity of *Alexandrium* varies depending on their growth stages and culture conditions, such as salinity, nutrients, and/or temperature [5,17,27,66,67,68] In general, high STXs content were detected under exponential phase of optimal growth conditions [7]. This was in accordance with our results that the highest toxicity was recorded in *A. catenella* Alex03 cultured at 16 °C. Perhaps, when cells divide rapidly in exponential phase, cell size may decrease with increase in the division rate. Hence, cell division decreases cellular contents, thereby decreasing their cellular toxicity.

Interestingly, we found that the STXs eq/cell of Alex03 was the highest at the optimum temperature 16 °C (maximum growth rate among three tests), and its mean cell size was much smaller than that at 12 °C. The escalation in total STXs eq at 16 °C was resulted by an increase in STX analogues at high TEF, particularly GTX1 [59]. The explanation that whether the toxicity of the cells is due to an increase in cell volume or STX production is controversial [37,67]. Previous results and our data, however, suggest that Alex03 should synthesize cellular STXs (e.g., GXT1) at low temperature, which supports optimal growth.

STXs eq and profiles of *Alexandrium* are partially explained by their biosynthesis gene regulation. As noted previously, *sxtA* and *sxtG* seem to be involved in early stage because they are considered as core genes linked to the initial process of STX production [28,45,47]. Thus, upregulation of the genes indicates the higher catalyzing rate of malonyl-CoA and arginine with increased Intermediate A’ and Intermediate B’ [34,69,70]. After this process, STX is then converted to GTX1–6 and C1–4 by other tailoring enzymes that are encoded by *sxt* cluster genes, resulting in STXs’ accumulation in toxic microorganisms [34]. In the present study, significant upregulations of both *sxtA4* and *sxtG* levels and STXs’ content were observed in *A. catenella* Alex03 exposed to cold shock, which showed the correlation of transcriptional responses and STX biosynthesis. This was also supported statistically by PCA, showing a positive relationship between *sxtA4* and *sxtG* expression levels and total STXs eq at low temperature and cold shock but not with heat shock. Similar to our results, *sxtA* expression levels and total STXs’ concentration decreased in cyanobacteria when exposed to 10 mM NaCl for 5 h [71]. Additionally, Geffroy et al. [72] suggested that transcriptional levels of *sxtA* were related to STXs’ content of toxic dinoflagellate *A. minutum*. In addition, both *sxtA4* and *sxtG* were positively correlated with total STXs’ production of *A. minutum* AmKB02 in different nutrient conditions [73]. On the other hand, results of weak correlation between mRNA levels of *sxtA* and *sxtG* and intracellular STXs have been reported in Mediterranean *A. minutum* [28]. The result indicates that STXs’ synthesis genes may be regulated by post-transcriptional regulation, suggesting the inconsistency between mRNA copies and its protein abundance [28,74]. However, since they significantly correlated in stoichiometric amounts [75,76], upregulations of STXs’ synthesis genes can lead to increase of the related proteins.

In contrast to cold temperature, we detected low toxicity and concentrations of diverse STX analogues from *A. catenella* Alex03 cultured at 20 °C and heat stress. Similarly, low toxins were measured at temperature higher than the optimum growth temperature; for example, 36.5 STXs eq µg/L at 23 °C and 83.3 STXs eq µg/L at 30 °C in the cyanobacterium *Aphanizomenon gracile* [19]. In addition, when *A. catenella* ACC02 was cultured at 10 to 16 °C, the lowest toxin levels (3.46 fmol STXs eq/cell) were measured at 16 °C [56]. In the present research, the concentrations of GTX1 and GTX4 and *sxtA4* expression levels decreased, while GTX3, STX, and neoSTX increased when *A. catenella* was exposed to heat stress. As mentioned above, downregulation of *sxtA4* and *sxtG* may reduce biosynthesis and accumulation of STXs [34,69]. *sxtG* mRNA levels increased in heat stress samples, and our results complemented the results of transcriptional responses and STX analogues. These results show that temperature regulates *sxtA4* expression levels, thereby affecting STXs eq/cell and profiles.

STX biosynthesis and modification are accomplished by many sxt enzymes, and their activity should be affected by temperature, resulting in different toxin levels [77]. For example, as one of the tailoring enzymes, N-sulfotransferase (sxtN) transfers the sulfate group (3′-phospho-adenosine-5′-phosphosulfate) into GTX2/3 in the dinoflagellate *Gymnodinium catenatum* [78]. In addition, the two putative sulfotransferases sxtN and sxtSUL produce the sulfated C-toxins in the cyanobacterium *Anabaena circinalis* [79,80]. These represent that sxtN is responsible for transferring STX into C-toxins (C1–4) and GTX-toxins (GTX1–4), changing the STXs profile of toxin-producing species [70]. Thus, sulfotransferase activity may alter STX profile of Alex03, and it was dependent on temperature, which can be predicted by the coefficient Q_10_ [81]. These results suggest that the sulfotransferase activity varies depending on the temperature, and may be more active at high temperature and heat shock in the toxic dinoflagellates *Alexandrium*.

## 4. Materials and Methods

### 4.1. Cell culture and Adaptation

The strain Alex03 (formerly known as LIMS-PS-2645) of *A. catenella* was obtained from the Marine Bio Resource Information System (MBRIS) of Korea Institute of Ocean Science & Technology (KIOST, Jangmok, Korea). It was isolated from the Korean South sea and cultured and maintained in f/2 medium without silicate [82] at 20 °C and 65 μmol photons/m^2^/s of photon flux density under a 12:12 h light–dark cycle.

For experiments related to temperature, cells cultured at 20 °C were adapted to each test temperature. In specific, we included low temperature cultures while gradually lowering the temperature (by 0.1 °C per day) using the standard cultures of 20 °C. Finally, three temperatures (12, 16, and 20 °C) were chosen to simulate the water temperature from March to May in the southern coasts of Korea (10–23 °C), because toxic *Alexandrium* blooms and PSP outbreaks were mostly reported during that season [49]. Each temperature-adapted culture in the exponential phase was sub-cultured in fresh f/2 medium for at least 6 months.

### 4.2. Design for Temperature Experiments

At the start of the experiment, each strain was inoculated at a density of 165.8 ± 6.3 cells/mL using fresh f/2 medium adapted to each temperature. The samples were either incubated at the same temperature or transferred to another incubator at different temperature for stress induction. To induce cold stress, the culture flasks incubated at 20 °C were transferred to 16 °C (20→16 °C) and 12 °C (20→12 °C), and from 16 °C to 12 °C (16→12 °C). Moreover, the cultures maintained at 12 °C were shifted to 16 °C (12→16 °C) or 20 °C (12→20 °C), and from 16 °C into 20 °C for inducing thermal stress. The samples were exposed to different temperatures for 72 h on day 15 and harvested on day 18.

### 4.3. Cell Density and Size Measurements

Cell counts were estimated for examining growth patterns and determined every two days using a plankton-counting chamber (Matsunami Glass, Osaka, Japan) under a light microscope (Carl Zeiss Axioskop, Oberkochen, Germany). All experiments were independently performed in triplicate. Individual cells in each culture were counted using Auto T4 CellometerTM (Nexcelom Biosciences, Lawrence, MA, USA) and were then used to calculate the average cell size.

### 4.4. DNA and RNA Extraction and cDNA Synthesis

Total RNA was extracted using 200 mL of *A. catenella* cultures with an initial density of 6.0 × 10^3^ cells/mL. In brief, the cells were harvested by centrifugation at 2000× *g* for 5 min at 4 °C, and the pellets were dissolved in 1 mL of TRIzol (Invitrogen, Carlsbad, CA, USA). To prevent RNA degradation, the samples were immediately frozen using liquid nitrogen and stored at −80 °C until RNA extraction.

For RNA extraction, the samples were physically lysed by freeze-thawing in liquid nitrogen and homogenized using zirconium beads (diameter 0.1 mm) using a Mini-bead beater (BioSpec Products Inc., Bartlesville, OK, USA). The samples were additionally purified using the Mini Spin Columns of the RNeasy Mini Kit (Qiagen, Valencia, CA, USA) as per the manufacturer’s instructions. After analyzing the RNA quality and quantity using Agilent 2100 Bioanalyzer (Agilent, Santa Clara, CA, USA), cDNA was synthesized using a TOPscript™ cDNA Synthesis Kit with random hexamer and oligo(dT)^18^ (Enzynomics, Daejeon, Korea) for gene cloning and gene expressional experiments. cDNA templates were diluted five times with nuclease-free water for molecular experiments.

Total genomic DNA (gDNA) was extracted from the *A. catenella* cells using the cetyltrimethylammonium bromide (CTAB) method [83].

### 4.5. Cloning of sxtA4, sxtG and 28S rRNA

Partial sequences of *sxtA4* and *sxtG* were retrieved from *A. catenella* EST data (157,342,442 sequence reads, 15.5 Gb) obtained in our laboratory, wherein the DNA sequences were determined using the Illumina Hiseq 2500 sequencing platform. These sequences were used to design primers for gene cloning. Nested PCR was performed using specific primer pairs (Table 2) and genomic DNA and cDNA templates. The reaction conditions for PCR were as follows: pre-denaturation at 95 °C for 5 min; followed by 35 cycles of 95 °C for 30 s, 55 °C for 30 s, 72 °C for 1 min and final extension at 72 °C for 10 min. PCR products were purified and cloned using the TOPcloner^TM^ TA kit (Enzynomics Inc., Daejeon, Korea). Each colony was used as template for PCR amplification and subjected to DNA sequencing.

### 4.6. Gene Characterization and Phylogenetic Analysis

The test species was identified by comparing the 28S rRNA sequence with that of other dinoflagellates obtained from GenBank and related researches [51]. The correct frames of two *sxt* genes were determined using BLASTx. The deduced amino acid sequences of *sxtA4* and *sxtG* were analyzed using the PROSITE-ExPASy Bioinformatics Resource portal (http://prosite.expasy.org/, accessed 4 January 2021) and the Interpro online tool (https://www.ebi.ac.uk/interpro/, accessed 4 January 2021) to identify the conserved domains.

Phylogenetic analyses were performed with sxt amino acid sequences of *A. catenella* Alex03 and related organisms. Each sequence matrix was properly aligned using Clustal X 2.0 (Conway Institute UCD Dublin, Dublin, Ireland) [86], and the final alignment of the sxtA4 and sxtG dataset consisted of 23 and 35 taxa and contained 236 and 256 amino acids, respectively. Phylogenetic tree derived from each dataset were constructed using the maximum likelihood (ML) algorithm, and the best-fitted model (LG+G) in MEGA X [87]. A bootstrap consensus tree inferred from 1000 replicates was used for taxa analysis.

### 4.7. Quantitative Real-Time PCR

Quantitative real-time PCR (qRT-PCR) was performed using the TOPreal qPCR 2× PreMIX SYBR Green Kit (Enzynomics Inc., Daejeon, Korea) in a CFX96 Real-Time PCR Detection System (Bio-Rad, Hercules, CA, USA). The reaction mixtures were prepared as follows: each of forward and reverse primer (10 pmol/μL; 1 μL), diluted cDNA (2 μL), TOPreal qPCR 2 × PreMIX buffer (SYBR Green with high ROX, 10 μL), and distilled water (6 μL). The reaction was performed as follows: 4 min at 50 °C; 10 min at 95 °C; followed by 40 cycles of 10 s at 95 °C, 15 s at 60 °C, and 15 s at 72 °C. Each reaction was performed in triplicate to calculate the mean value. Specificity of amplification was identified by the melting curve generated by heating the sample from 65 °C to 95 °C. Primer efficiency was calculated from the standard curve using the threshold cycle (Ct) values for a 10-fold dilution series of the cDNA. Among reference genes, *α tubulin* (*TUA*) was used as internal controls for data normalization. Results were analyzed using the Student’s t-test in SPSS software (Version 19.0; IBM Corp., Armonk, NY, USA).

### 4.8. High-Pressure Liquid Chromatography-Fluorescence Detection (HPLC-FLD)

We harvested *A. catenella* from 100 mL of culture by centrifugation at 2000× *g* for 10 min. The pellets were immediately resuspended in 0.01 M HCl (pH 3.0) and homogenized using a bead crusher (Taitec Corporation, Nishikata, Japan). The homogenized samples were boiled at 95 °C for 5 min. All samples were filtered through a 0.2-micrometer GVS syringe filter (GVS, Bologna, Italia) to remove cell debris.

Standard solutions including GTX1-4,6, C1-2, dcGTX2-3, STX, neoSTX, and dcSTX purchased from National Research Council Canada (NRC; Halifax, NS, Canada). STXs’ analysis was performed using a HPLC-FLD system (Waters, Milford, MA, USA) and the post-column method with a slight modification of the process described by Rey et al. [88]. The fluorescence detector operated at an excitation wavelength of 330 nm and emission was scanned at 390 nm. STX analogues were separated using a Hypercarb^®^ column (150 mm × 2.1 mm i.d., 5 µm; Thermo Scientific, Madrid, Spain) and quantified using a 5-point calibration curve prepared using the reference standards. The concentrations of total STXs were calculated as STXs equivalent per cells (STXs eq fmol/cell) by referring to the TEF. Each STX analog has a different TEF value and the total STXs eq calculation was based on FAO/WHO [59]. The TEF sets the toxicity of STX to 1 and calculates the relative toxicity of the other derivatives.

### 4.9. Statistical Analysis and Principal Component Analysis (PCA)

Statistical analyses were performed using the SPSS statistical package (Version 19.0; IBM Corp., Armonk, NY, USA). Mean and standard errors were calculated for each treatment, and significant differences were determined with one-way analysis of variance (ANOVA), followed by the Student–Newman–Keuls multiple comparisons test. Probability (p) values of one-way ANOVA tests were indicated as * *p* < 0.05, ** *p* < 0.01, and *** *p* < 0.001. *p* < 0.05 was considered as statistically significant.

In addition, principal component analysis (PCA) was performed to evaluate the relationship among tested variables, such as water temperatures, cell size, and STX biosynthesis (total STXs eq and relative expression of *sxtA4* and *sxtG*) using the Paleontological Statistics package (Past v.4.03; Natural History Museum, Blindern, Norway).

## 5. Conclusions

**Figure 6:**
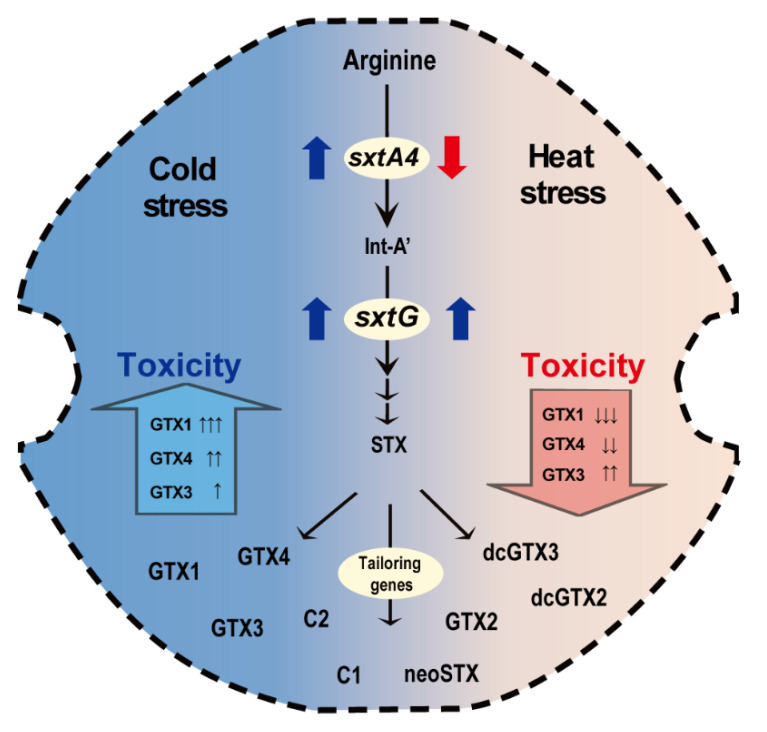
A schematic representation of the putative saxitoxin metabolic pathway in *Alexandrium catenella.* The transcriptional levels of the core genes *sxtA4* and *sxtG* regulate STX biosynthesis and are altered by water temperature. Cold stress upregulated both *sxtA4* and *sxtG*, leading to higher STX production, whereas heat stress downregulated *sxtA4* to lower the toxicity. Various analogues were analyzed.

This work first reported STXs’ toxicity and contents in the toxic dinoflagellate *A. catenella* Alex03 under different temperatures and the possible relationships with the regulation of two core genes *sxtA4* and *sxtG*. The optimal temperature for Alex03 was 16 °C, exhibiting the highest growth rate and cellular STXs’ content. When Alex03 was exposed to low temperatures and cold stress, the total STXs eq increased notably with increased GTX1 and *sxtA4* and *sxtG* transcriptional levels (Figure 6). Conversely, thermal shock lowered STXs eq/cell, but a wider variety of STX analogues were detected with downregulation of *sxtA4*. Statistical analysis demonstrated that *sxtA4* expression was correlated with STXs eq in low temperature and cold shock samples, whereas *sxtG* was sensitive to temperature changes. These results were in accordance with the results of field monitoring data, i.e., most PST contamination of shellfish by *A. catenella* occurred in spring and autumn (8.2–15 °C) in Korean coasts [89]. In addition, resting cysts of the *Alexandrium* spp. germinated in November at 19.1–19.6 °C [50], and optimal growth temperature for *A. catenella* was recorded at 10–20 °C. Based on these field data and the results, we could conclude that temperature affected the STXs and the regulation of their biosynthesis genes to the highest level at optimal and cold temperatures in *A. catenella*. STX biosynthesis and modification involves many enzymes and their functional gene regulations, and thus, further researches are necessary to understand the whole transcriptional responses of STX synthesis genes in the future.

## Figures and Tables

**Table 1:** Toxin production in the genus *Alexandrium* under different temperatures from published literatures.

Species	Strain	Temperature	Toxins	STXs eq (fmol/cell)	Reference
*Alexandrium catenella*	ACC02	10–16 °C	electrophysiological test	3.427.7	[56]
	CCAP1119/27	15 °C	STX, neoSTX, dxSTX, GTX1-6, C1–2, C4	2732.5 fg STXs eq/cell	[60]
	ATTL01 ATTL02	15 °C	GTX 1,4,5, C1–2	5.3–44.3 fg STXs eq/cell	[22]
	BAH91	15 °C	STX, B1-2, C1–2	9.9	[61]
	Otsuchi Bay isolated	15 °C	STX, neoSTX, GTX1, C1–2	34.5	[62]
	ACT03	10–30 °C	GTX3-5, C1–4	2.9–50.3	[58]
*Alexandrium fundyense*	BOF	5–20 °C	GTX1–4, STX, neoSTX	211–544	[61]
	MI	5–20 °C	GTX1–4, STX, neoSTX	100–532	[61]
*Alexandrium tamrense*	BAH181	15 °C	GTX1–4, neoSTX, STX, B1–2, C1–2	42.3	[63]
	GTPP01	15 °C	GTX1–4, neoSTX, STX, B1–2, C1–2	33.4	[63]
	ATHS-95	17 °C	GTX1–4, C1–4	1.35–2.7	[64]
*Alexandrium minutum*	AmSp01	25 °C	GTX1, 3, 4, neoSTX	11.2–12.8	[17]
	AmSp03	25 °C	GTX1, 4, neoSTX	9.1–11.8	[17]
	AmSp04	25 °C	GTX1, 3, 4, neoSTX	5.1–11.2	[17]
	AmSp05	25 °C	GTX1–4, neoSTX, dcSTX,	3.0–9.5	[17]
	AmSp17	25 °C	GTX1, 3, 4, dcSTX, neoSTX	5.6–6.3	[17]
	AL3T	15 °C	GTX1–4	3	[63]
*Alexandrium lusitanicum*	BAH91	15 °C	GTX1–4	16	[63]
*Alexandrium affine*	AABCV-1	15–34 °C	non-toxic	non-toxic	[65]
	CCMP112	16–20 °C	non-toxic	non-toxic	[3]
	CS 312/02	16–20 °C	non-toxic	non-toxic	[3]
*Alexandrium andersonii*	CCMP1597	16–20 °C	non-toxic	non-toxic	[3]
	CCMP2222	16–20 °C	non-toxic	non-toxic	[3]

**Table 2:** Primers used in this study.

Gene	Primer	Nucleotide Sequence (5’→3’)	Remark	Source
*sxtA4*	Sxt007F	ATGCTCAACATGGGAGTCATCC	ORF	[45]
	Sxt008R	GGGTCCAGTAGATGTTGACGATG	ORF	[45]
	sxtA4qF	GAGCAACCCTTCGGGTATGGT	qRT-PCR	This study
	sxtA4qR	TCAGAATGCCGAACTTCTCGTCG	qRT-PCR	This study
*sxtG*	sxtG001F	GCCGATGTATGACTTCTACAAGAG	ORF	This study
	sxtG002F	CATCCCAGACTGGTACATGC	ORF	This study
	sxtG001R	CCGTATGGATGTACCTGTGC	ORF	This study
	sxtG002R	AGAGCGTGTTCAAGTGGTAGC	ORF	This study
	sxtGqF	GGACATGGACGAGAATAGCTG	qRT-PCR	This study
	sxtGqR	GATGGCGAGCACGTTTATGC	qRT-PCR	This study
*α-tubulin*	TUA qF	CTTCCAGGGCTTCATGGTG	qRT-PCR	This study
	TUA qR	AGACACGTTTGGCTCCTG	qRT-PCR	This study
*Actin*	ACT-US-408-F	ACTTGATTTGCTTGGTGGGAG	qRT-PCR	[84]
	ACT-US-645-R	AAGTCCAAGGAAGGAAGCATC	qRT-PCR	[84]
*28S rRNA*	28F01	CCGCTGAATTTAAGCATATAAGTAAGC	rRNA	[85]
	28R691	CTTGGTCCGTGTTTCAAGAC	rRNA	[85]

## Data Availability

The data presented in this study are available on request from the corresponding authors. In addition, the data that support the findings of this study are openly available in GenBank with the accession numbers MW882944, MW884258, and MW884259.
